# Grain size dependent photoresponsivity in GaAs films formed on glass with Ge seed layers

**DOI:** 10.1038/s41598-021-89342-w

**Published:** 2021-05-12

**Authors:** T. Nishida, K. Igura, T. Imajo, T. Suemasu, K. Toko

**Affiliations:** 1 Institute of Applied Physics, University of Tsukuba, 1-1-1 Tennodai, Tsukuba, Ibaraki 305-8573 Japan; 2 JSPS Research Fellow, 8 Ichiban-cho, Chiyoda-ku, Tokyo, 102-8472 Japan; 3 PRESTO, Japan Science and Technology Agency, 4-1-8 Honcho, Kawaguchi, Saitama 332-0012 Japan

**Keywords:** Applied physics, Electrical and electronic engineering, Semiconductors, Electronic devices

## Abstract

The strong correlation between grain size and photoresponsivity in polycrystalline GaAs films on glass was experimentally demonstrated using Ge seed layers with a wide range of grain sizes (1‒330 μm). The crystal evaluations using Raman spectroscopy, scanning electron microscopy, electron backscatter diffraction, and transmission electron microscopy revealed that 500-nm-thick GaAs films epitaxially grown from the Ge seed layers at 550 °C inherited the grain boundaries and crystal orientations in Ge. With increasing grain size, the photoresponsivity corresponding to GaAs increased from 0.01 to 3 A W^−1^ under a bias voltage of 0.3 V. The maximum value approached that of the GaAs film formed simultaneously on a single-crystal Ge wafer, indicating the high potential of the large-grained GaAs film. Knowledge gained from this study will be essential for designing advanced solar cells based on polycrystalline III–V compound semiconductors using inexpensive substrates.

## Introduction

The conversion efficiency of solar cells using III–V compound semiconductors has exceeded 47.1% for multijunctions and 30.5% for single-junction structures^1^. However, the high fabrication cost of these solar cells has limited their applications, e.g., for space applications. Replacing single-crystal wafers with an inexpensive substrate can reduce the fabrication cost^2, 3^.

The transfer technique is an effective method for developing high-efficiency solar cells on glass or flexible substrates; however, the technique is not cost effective because it begins with a single-crystal wafer^4–6^. Therefore, the direct synthesis of polycrystalline (poly-) GaAs layers on inexpensive substrates has been vigorously investigated for decades. Because the grain boundaries in poly-GaAs serve as recombination centers, the larger grain size (GS) provides a higher conversion efficiency of the solar cell^7, 8^. Theoretically, the conversion efficiency of a poly-GaAs thin-film solar cell is estimated to be 22%, with a GS of 100 μm^8^. To fabricate large-grained GaAs films on inexpensive substrates, researchers have investigated various techniques, including vacuum deposition^9–15^, crystallization of amorphous films^16–19^, and chemical synthesis^20, 21^. However, no photoresponse has been obtained owing to difficulty in synthesizing large-area GaAs layers with large grains (> 1 μm). Therefore, the correlation between GS and photoresponsivity in poly-GaAs layers has never been experimentally demonstrated due to the difficulty of controlling the GS of the poly-GaAs.

Ge has been used as an ideal epitaxial template for GaAs layers because its lattice parameter matches that of GaAs^22–24^. Therefore, the formation of a large-grained Ge thin film on an inexpensive substrate and epitaxially grown GaAs has been widely investigated^25–27^. Recently, a conversion efficiency of 11.5% was demonstrated by sequentially growing Ge and GaAs films on a biaxially textured oxide layer formed on a metal tape^28^. Considering that the GS of the GaAs film is only a few micrometers, its enlargement will further improve the efficiency.

Previously, we investigated the synthesis of poly-Ge thin films on insulating substrates. In the solid-phase crystallization (SPC) of amorphous (a-) Ge, the GS of the resulting poly-Ge was controlled over a wide range (10 nm‒20 μm) by modulating the deposition temperature and annealing temperature^29^, inserting an underlayer^30^, and adding certain elements (Sn, Sb, and As)^31–33^. Furthermore, the layer exchange technique^34, 35^ allowed us to form a large-grained (> 100 μm) Ge seed layer^36, 37^, which provided a large-grained poly-GaAs layer with high photoresponsivity^38, 39^. Using the recent GS control technology of poly-Ge, we controlled the GS of the poly-GaAs layer over a wide range (1‒330 μm) and experimentally demonstrated the correlation between the GS of poly-GaAs and its photoresponse property.

## Experimental

**Figure 1:**
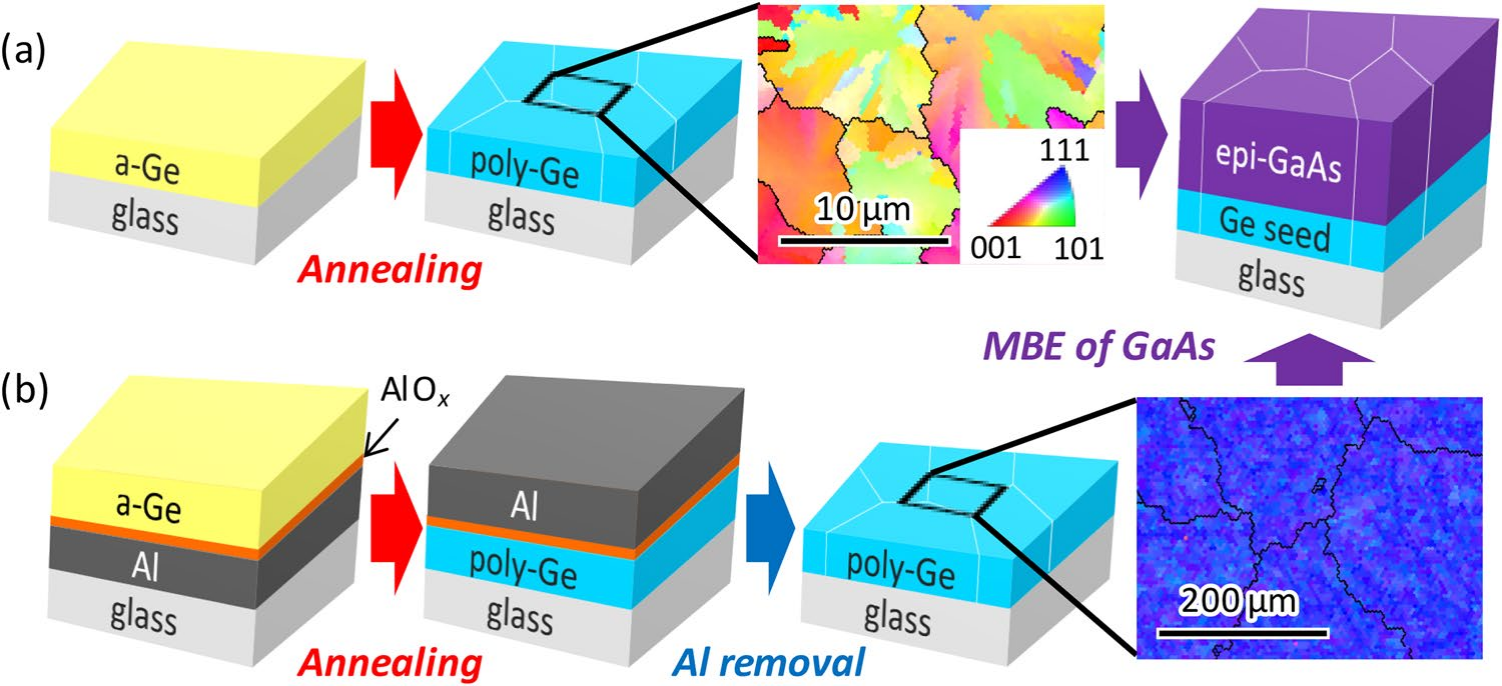
Schematic of sample preparation. (**a**) Samples in Groups A–C using SPC-Ge seed layers and (**b**) samples in Group D using ALILE-Ge seed layers. Representative crystal orientation maps of SPC-Ge (Sb-doped, *T*_anneal_ = 375 °C) and ALILE-Ge (*t*_AE_ = 3 min) are shown in expanded views, where color scheme indicates crystal orientation (refer to legend in inset). Black solid lines in crystal orientation maps show grain boundaries surrounding grains.

Ge seed layers with various GSs were prepared on a quartz glass (SiO_2_) substrate using undoped^29^ or impurity-doped SPC^32, 33^ and Al-induced layer exchange (ALILE)^36, 37^. In the SPC process (Fig. 1a), 200-nm-thick amorphous Ge (a-Ge) precursors were prepared by heating the samples at 125 °C to densify the a-Ge. Ge atoms were supplied by Knudsen cells of a molecular beam deposition system (base pressure: 5 × 10^−7^ Pa) for 200 min at a deposition rate of 1 nm min^−1^. Subsequently, the samples were loaded into a conventional tube furnace in a N_2_ (99.9%) atmosphere and annealed at annealing temperature *T*_anneal_ = 375–450 °C to induce SPC. In the ALILE process (Fig. 1b), 50-nm-thick Al and 70-nm-thick a-Ge thin films were prepared at room temperature using DC magnetron sputtering (base pressure: 5 × 10^−5^ Pa) with Ar plasma. Between the two deposition cycles, the Al film was exposed to air for *t*_AE_ = 1–10 min to form a native Al oxide (AlO_*x*_) layer. The sample was annealed at 350 °C for 50 h under a N_2_ atmosphere to induce layer exchange between the Al and Ge layers. After annealing, the sample was treated using a H_2_O_2_ (50%) solution to remove Ge islands followed by a HF solution (1.5%) to remove the Al and AlO_*x*_ layers^37^. The resulting Ge layer is highly p-doped (3 × 10^20^ cm^−3^) due to the Al atoms in Ge which work as acceptors. Subsequently, the 500-nm-thick GaAs layer was grown on each Ge seed layer at 550 °C using molecular beam epitaxy (base pressure: 1 × 10^−7^ Pa), where Ga and As atoms were supplied by Knudsen cells for 2.5 h at a deposition rate of 200 nm h^−1^ with an As/Ga beam flux ratio of 294. We note that the As pressure was 1 × 10^−2^ Pa, which was an order of magnitude higher than our previous reports^38, 39^ and was effective in improving the photoresponsivity.

The detailed characteristics of the Ge seed layers are summarized in Table 1. To control the GS of Ge, Sb (2.8 × 10^20^ cm^−3^) and As (concentration *C*_As_ = 1.0 × 10^19^–5.9 × 10^20^ cm^−3^) were added in a-Ge using Knudsen cells. The Sb and As influence the growth kinetics of Ge and act not only as dopants but also as surfactants: the growth rate and the saturated grain size significantly depend on the dopant concentration because impurity doping promotes the migration of semiconductor atoms^32, 33^. The dopant atoms fully remain in Ge without segregation and some are activated to provide carriers. Group A corresponds to undoped SPC-Ge^29^; Group B to Sb-doped SPC-Ge^32^; Group C to As-doped SPC-Ge^33^; and Group D to ALILE-Ge^37^. To control GS within a wide range, for each group, the GS was controlled by modulating parameters such as *T*_anneal_ (375–450 °C), *C*_As_ (1.0 × 10^19^–5.9 × 10^20^ cm^−3^), and *t*_AE_ (1–10 min). The sheet resistance of the Ge layers, measured using the van der Pauw method, varies for each sample owing to the difference in the carrier concentration and carrier mobility. In the photoresponsivity measurement, the difference in sheet resistance was negligible because of the much higher resistance of semi-insulating undoped GaAs. Through these processes, the GS of the Ge layers, which was determined via electron backscatter diffraction (EBSD), as representatively shown in Fig. 1, was controlled over a wide range (1–330 μm). For comparison, we grew GaAs layers on a bare SiO_2_ glass substrate and a single-crystal (sc-) Ge(111) wafer (resistivity < 10^−2^ Ω cm).

**Table 1:** Properties of Ge seed layers.

Group	Crystallization process	Thickness (nm)	Parameter to control GS	Range of average GS (μm)	Sheet resistance (Ω/□)	Ref
A	Undoped SPC	200	*T*_anneal_ (375–450 °C)	1.2–5.7	2130–2670	29
B	Sb-doped SPC	200	*T*_anneal_ (375–450 °C)	6.3–12.9	840–1420	32
C	As-doped SPC	200	*C*_As_ (1.0 × 10^19^–5.9 × 10^20^ cm^−3^)	3.5–28.3	170–2860	33
D	ALILE	50	*t*_AE_ (1–10 min)	145–330	140–260	37

## Results and discussion

**Figure 2:**
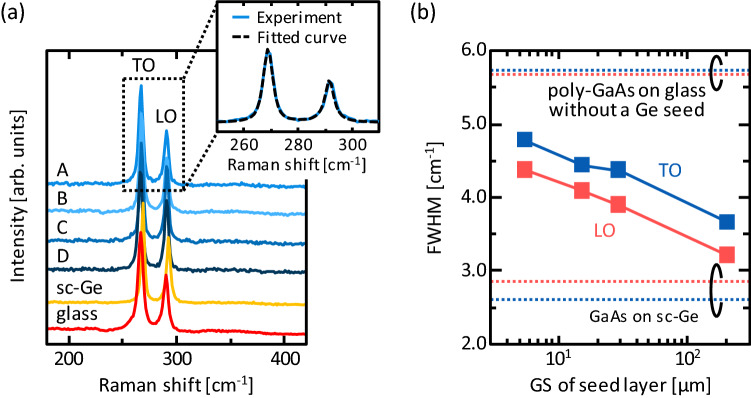
Raman spectroscopy study. (**a**) Raman spectra of samples in Group A (*T*_anneal_ = 375 °C), Group B (*T*_anneal_ = 375 °C), Group C (*C*_As_ = 1.2 × 10^20^ cm^−3^), and Group D (*t*_AE_ = 3 min). Fitting result of Raman spectrum shown representatively in expanded view. (**b**) FWHMs of TO and LO mode Raman peaks for samples in Groups A–D. Data for GaAs layers grown on sc-Ge(111) wafer and glass without Ge seed layer are shown for comparison.

To evaluate the effects of the GS of Ge seed layers on the crystallinity of GaAs, we selected typical samples from each group: *T*_anneal_ = 375 °C for Group A and B, *C*_As_ = 1.2 × 10^20^ cm^−3^ for Group C, and *t*_AE_ = 3 min for Group D. The crystal quality of the grown GaAs layers was investigated using Raman spectroscopy (JASCO NRS-5100, spot diameter 20 μm, wavelength 532 nm). As shown in Fig. 2a, all samples exhibit sharp peaks in the Raman spectra, corresponding to the transverse optical (TO) mode (~ 270 cm^−1^) and longitudinal optical (LO) mode (~ 290 cm^−1^) of crystalline GaAs^13, 16^. The intensity ratio of the TO and LO peaks are almost the same for all samples. To analyze the full width at half maximum (FWHM) of the crystalline GaAs peaks, each spectrum was fitted, as shown in Fig. 2a. The peak fitted sufficiently well for correct FWHM calculation. Figure 2b shows that the FWHMs of both the TO and LO peaks decrease with increasing GS of the Ge seed layers and approach to those of the GaAs layer formed on a sc-Ge(111) wafer. For all samples, the FWHMs are lower than that of a GaAs layer directly formed on glass without a Ge seed layer. These results suggest that the larger GS of the Ge seed layer provides higher crystallinity in the GaAs layer.

**Figure 3:**
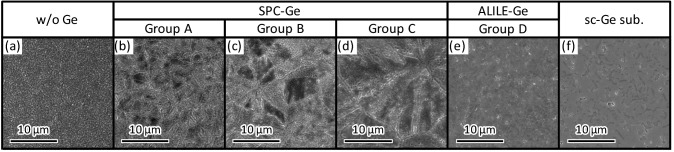
Surface SEM images of GaAs layers. (**a**) GaAs layer grown on glass without Ge seed layer, (**b**) sample in Group A (*T*_anneal_ = 375 °C), (**c**) sample in Group B (*T*_anneal_ = 375 °C), (**d**) sample in Group C (*C*_As_ = 1.2 × 10^20^ cm^−3^), (**e**) sample in Group D (*t*_AE_ = 3 min), and (**f**) GaAs layer grown on sc-Ge wafer. The samples were wet etched for 10 min to enhance grain boundaries and reveal GS.

To clarify the grain boundaries in GaAs, we dipped the samples in HNO_3_:H_2_O (1:3) solution for 10 min and observed the surface morphology using scanning electron microscopy (SEM). Figure 3a suggests that the GaAs layer grown without a Ge seed layer comprises submicron grains. Figure 3b–d show that the samples in Groups A–C contain grain boundaries in the GaAs layers, whose density varied with the samples. The contrasts in the SEM images are likely due to the electron channeling effect^40^: a phenomenon in which the penetration depth of an electron beam changes depending on the crystal orientation. Conversely, Fig. 3e shows that the sample in Group D contains no grain boundaries in the observed region and exhibits a relatively similar surface to the GaAs layer grown on an sc-Ge(111) wafer (Fig. 3f). This behavior is likely due to the epitaxial growth of GaAs from the large-grained (111)-oriented Ge seed layer.

**Figure 4:**
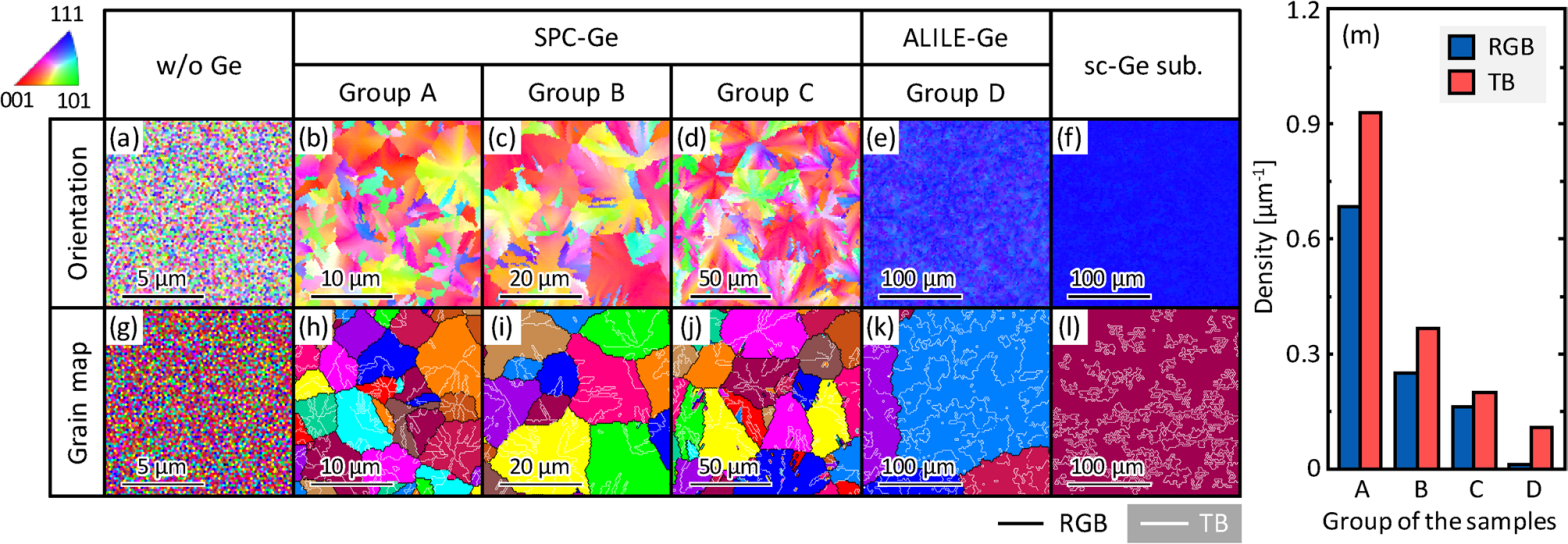
EBSD analyses of GaAs layers. (**a**–**f**) Crystal orientation and (**g**–**l**) grain maps of (**a**, **g**) GaAs layer grown on glass without Ge seed layer, (**b**, **h**) sample in Group A (*T*_anneal_ = 375 °C), (**c**, **i**) sample in Group B (*T*_anneal_ = 375 °C), (**d**, **j**) sample in Group C (*C*_As_ = 1.2 × 10^20^ cm^−3^), (**e**, **k**) sample in Group D (*t*_AE_ = 3 min), and (h,l) GaAs layer grown on sc-Ge(111) wafer. Color in crystal orientation maps indicates crystal orientation (refer to legend in inset). Each colored area in grain maps corresponds to one grain. Solid black lines represent random grain boundaries (RGBs) surrounding grains, and white solid lines represent twin boundaries (TBs). (**m**) Density of RGBs and TBs determined by grain maps shown in (**h**–**k**).

Figure 4a‒f show the crystal orientation maps of the representative GaAs layers. The GS of the sample without a Ge seed layer is below the detection limit of the EBSD system (Fig. 4a). The samples in Groups A–C with the SPC-Ge seed layers are randomly oriented (Fig. 4b‒d), whereas the sample in Group D is highly (111)-oriented (Fig. 4e). The crystal orientation maps are similar to those of each Ge seed layer (Fig. 1)^29, 32, 33, 37^. The GaAs grown on the sc-Ge(111) wafer is a (111)-oriented single crystalline (Fig. 4f). These results suggest the epitaxial growth of GaAs from Ge. To determine the GS of GaAs, the crystal orientation maps were converted to grain maps, as shown in Fig. 4g‒l, where a grain was defined as an area surrounded by random grain boundaries (RGBs). The average GS of the samples was determined to be 5.4 μm for Group A (Fig. 4h), 15.2 μm for Group B (Fig. 4i), 27.8 μm for Group C (Fig. 4j), and 244 μm for Group D (Fig. 4k). Furthermore, the grain maps show that the poly-GaAs layers (Fig. 4h‒k) and the sc-GaAs layer (Fig. 4l) contains twin boundaries (TBs). This suggests that some TBs have inherited Ge^41^ and have been introduced during the growth of GaAs. Figure 4m shows that the density of the RGBs and TBs in GaAs depends significantly on the seed layer. A larger GS provided a lower density of RGBs and TBs. Hence, poly-GaAs films with significantly different grain boundary densities were formed by controlling the GS of the Ge seed layers.

**Figure 5:**
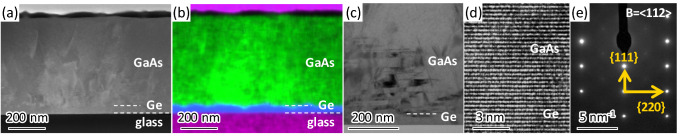
Cross-section characterization of sample in Group D (*t*_AE_ = 3 min). (**a**) High-angle annular dark-field scanning TEM image. (**b**) EDX elemental map. (**c**) Bright-field TEM image. (**d**) High-resolution lattice image showing GaAs/Ge interface. (**e**) SAED pattern obtained from region including GaAs and Ge layers with selected area of diameter 800 nm.

We evaluated the detailed cross-sectional structure of the sample in Group D using a transmission electron microscopy (TEM; FEI Tecnai Osiris) operating at 200 kV, equipped with an energy-dispersive X-ray spectrometer (EDX). Figure 5a, b show the stacked structure of GaAs/Ge/glass. Figure 5c shows that the GaAs film contains stacking faults. Anti-phase boundaries were not clearly observed likely because the GaAs on Ge(111) preferentially has type A polarity and does not form anti-phase domains^42, 43^. Considering that the Ge seed layer was free of extended defects^37^, the defects in GaAs are presumed to be caused by the rough surface of the ALILE-Ge layer and/or the growth instability of GaAs on (111) surfaces. These problems can be solved by introducing a GaAs buffer layer and/or optimizing the growth conditions, such as the As flux and growth rate of GaAs. Figure 5d shows that the (111) planes are in an orderly line from the Ge to GaAs films. Because the GaAs/Ge interface is continuous, it is difficult to identify from the lattice image. The selected-area electron diffraction (SAED) pattern shown in Fig. 5e, including Ge and GaAs, indicates a (111)-oriented single crystalline pattern (800 nm in diameter). The SAED spots of Ge and GaAs overlap because their lattice constants are close (approximately 0.2% mismatch). These results indicate that the GaAs film epitaxially grew from the Ge seed layer.

**Figure 6:**
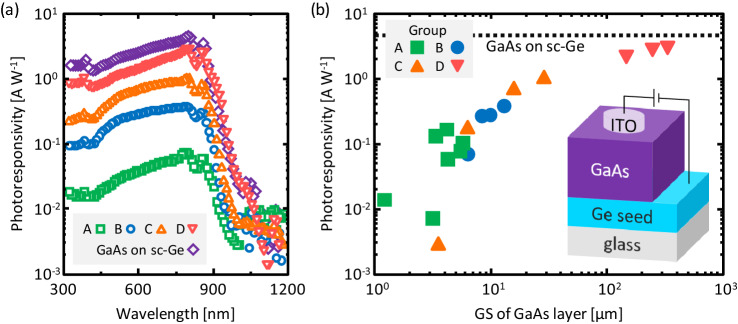
Photoresponse properties of samples in Groups A–D, where bias voltage was 0.3 V. (**a**) Photoresponsivity of samples in Group A (*T*_anneal_ = 375 °C), Group B (*T*_anneal_ = 375 °C), Group C (*C*_As_ = 1.2 × 10^20^ cm^−3^), and Group D (*t*_AE_ = 10 min). (**b**) Maximum photoresponsivity values for each spectrum as a function of GS. Data for GaAs on sc-Ge(111) wafer shown for comparison. Inserted image shows schematic diagram of sample for measurement.

After preparing circular ITO electrodes (diameter: 1 mm), the photoresponsivity was measured for all samples (1 cm^2^ square size) with the structure illustrated in Fig. 6. Standard AM1.5, 100 mA cm^−2^ illumination was applied onto an ITO electrode using a shading mask. A bias voltage of 0.3 V was applied to the ITO electrode with respect to the Ge seed layer. Because a PN junction was absent in the GaAs of the current sample, the photogenerated electrons in GaAs were transferred to the surface ITO electrode via drift in photoconductivity mode. Figure 6a shows clear photoresponse spectra rising near a wavelength of 900 nm, corresponding to the bandgap of GaAs for each sample. The photoresponsivity is maximum at wavelengths of 700–800 nm for all samples and depends significantly on the seed layer type. The maximum photoresponsivity values for each spectrum are summarized in Fig. 6b as a function of the GS of GaAs. The photoresponsivity increases with the GS and approaches that of the sc-GaAs layer. This behavior is likely due to the decrease in the recombination rate of carriers at the grain boundaries with increasing GS, consistent with theoretical calculations of conversion efficiency of GaAs thin-film solar cells^7, 8^. A maximum photoresponsivity of 3.0 A W^−1^ was obtained for the sample with a GS of 330 μm.

## Conclusions

The crystal evaluations using Raman spectroscopy, SEM, EBSD, and TEM revealed that the crystallinity and GS of GaAs film grown on a glass substrate could be modulated considerably using Ge seed layers with a wide range of GSs. The strong correlation between the GS and photoresponsivity of poly-GaAs films was demonstrated: the photoresponsivity increased from 0.01 to 3 A W^−1^ when the GS increased from approximately 1 to 330 μm. The maximum photoresponsivity was approximately that of the GaAs film simultaneously formed on a single-crystal Ge wafer, indicating the high potential of the large-grained GaAs film. Although there are still many challenges including the formation of PN junctions for poly-GaAs, the knowledge gained in this study will be useful for designing advanced solar cells based on polycrystalline III–V compound semiconductors using inexpensive substrates.

## References

[1] Green M (2021). Solar cell efficiency tables (version 57). Prog. Photovolt. Res. Appl..

[2] Venkatasubramanian R, Siivola E, O’Quinn B, Keyes B, Ahrenkiel R (1997). Pathways to high-efficiency GaAs solar cells on low-cost substrates. AIP Conf. Proc..

[3] Yoon J (2010). GaAs photovoltaics and optoelectronics using releasable multilayer epitaxial assemblies. Nature.

[4] Shahrjerdi D (2012). High-efficiency thin-film InGaP/InGaAs/Ge tandem solar cells enabled by controlled spalling technology. Appl. Phys. Lett..

[5] Onyegam EU (2013). Exfoliated, thin, flexible germanium heterojunction solar cell with record FF=58.1%. Sol. Energy Mater. Sol. Cells.

[6] Moon S, Kim K, Kim Y, Heo J, Lee J (2016). Highly efficient single-junction GaAs thin-film solar cell on flexible substrate. Sci. Rep..

[7] Yamaguchi M, Itoh Y (1986). Efficiency considerations for polycrystalline GaAs thin-film solar cells. J. Appl. Phys..

[8] Kurtz SR, McConnell R (1997). Requirements for a 20%-efficient polycrystalline GaAs solar cell. AIP Conf. Proc..

[9] Yang JJJ, Dapkus PD, Dupuis RD, Yingling RD (1980). Electrical properties of polycrystalline GaAs films. J. Appl. Phys..

[10] Tsuji S, Iri E, Takakura H (1992). Electronic properties of GaAs thin film deposited on silica substrate by RF sputtering. Jpn. J. Appl. Phys..

[11] Mochizuki K, Nakamura T, Mishima T, Masuda H, Tanoue T (1994). Molecular beam deposition of low-resistance polycrystalline GaAs. J. Electron. Mater..

[12] Imaizumi M (2000). Low-temperature growth of GaAs polycrystalline films on glass substrates for space solar cell application. J. Cryst. Growth.

[13] Erlacher A (2006). Texture and surface analysis of thin-film GaAs on glass formed by pulsed-laser deposition. J. Non. Cryst. Solids.

[14] Kajikawa Y, Okuzako T, Takami S, Takushima M (2010). Electrical properties of polycrystalline GaInAs thin films. Thin Solid Films.

[15] Şenay V, Özen S, Pat S, Korkmaz Ş (2016). Optical, structural, morphological and compositional characterization of a Co-doped GaAs semiconducting thin film produced by thermionic vacuum arc. J. Alloys Compd..

[16] Campomanes, R. R., Dias da Silva, J. H., Vilcarromero, J. & Cardoso, L. P. Crystallization of amorphous GaAs films prepared onto different substrates. *J. Non. Cryst. Solids* **299**–**302**, 788–792 (2002).

[17] Epple JH (2003). Formation of highly conductive polycrystalline GaAs from annealed amorphous (Ga, As). J. Appl. Phys..

[18] Pirzada D, Cheng GJ (2009). Microstructure and texture developments in multiple pulses excimer laser crystallization of GaAs thin films. J. Appl. Phys..

[19] Saito M (2021). Solid-phase crystallization of gallium arsenide thin films on insulators. Mater. Sci. Semicond. Process..

[20] Nayak J, Sahu SN (2001). Study of structure and optical properties of GaAs nanocrystalline thin films. Appl. Surf. Sci..

[21] Sathasivam S (2014). Solution processing of GaAs thin films for photovoltaic applications. Chem. Mater..

[22] Yamaguchi M, Takamoto T, Araki K, Ekins-Daukes N (2005). Multi-junction III–V solar cells: current status and future potential. Sol. Energy.

[23] King RR (2007). 40% efficient metamorphic GaInP∕GaInAs∕Ge multijunction solar cells. Appl. Phys. Lett..

[24] Kajikawa Y (2017). Suppression of twin generation in the growth of GaAs on Ge(111) substrates. J. Cryst. Growth.

[25] Mauk MG, Balliet JR, Feyock BW (2003). Large-grain (>1-mm), recrystallized germanium films on alumina, fused silica, oxide-coated silicon substrates for III–V solar cell applications. J. Cryst. Growth.

[26] Tsao C-Y, Huang J, Hao X, Campbell P, Green MA (2011). Formation of heavily boron-doped hydrogenated polycrystalline germanium thin films by co-sputtering for developing p^+^ emitters of bottom cells. Sol. Energy Mater. Sol. Cells.

[27] Pelati D (2020). Microstructure of GaAs thin films grown on glass using Ge seed layers fabricated by aluminium induced crystallization. Thin Solid Films.

[28] Khatiwada D (2020). High-efficiency single-junction p-i-n GaAs solar cell on roll-to-roll epi-ready flexible metal foils for low-cost photovoltaics. Prog. Photovolt. Res. Appl..

[29] Toko K, Yoshimine R, Moto K, Suemasu T (2017). High-hole mobility polycrystalline Ge on an insulator formed by controlling precursor atomic density for solid-phase crystallization. Sci. Rep..

[30] Imajo T, Moto K, Yoshimine R, Suemasu T, Toko K (2019). High hole mobility (≥500 cm^2^ V^−1^ s^−1^) polycrystalline Ge films on GeO_2_-coated glass and plastic substrates. Appl. Phys. Express.

[31] Moto K, Yoshimine R, Suemasu T, Toko K (2018). Improving carrier mobility of polycrystalline Ge by Sn doping. Sci. Rep..

[32] Takahara D, Moto K, Imajo T, Suemasu T, Toko K (2019). Sb-doped crystallization of densified precursor for n-type polycrystalline Ge on an insulator with high carrier mobility. Appl. Phys. Lett..

[33] Saito M, Moto K, Nishida T, Suemasu T, Toko K (2019). High-electron-mobility (370 cm^2^/Vs) polycrystalline Ge on an insulator formed by As-doped solid-phase crystallization. Sci. Rep..

[34] Toko K, Suemasu T (2020). Metal-induced layer exchange of group IV materials. J. Phys. D. Appl. Phys..

[35] Wang ZM, Wang JY, Jeurgens LPH, Phillipp F, Mittemeijer EJ (2008). Origins of stress development during metal-induced crystallization and layer exchange: annealing amorphous Ge/crystalline Al bilayers. Acta Mater..

[36] Toko K (2014). Low-temperature (180 °C) formation of large-grained Ge(111) thin film on insulator using accelerated metal-induced crystallization. Appl. Phys. Lett..

[37] Toko K, Nakazawa K, Saitoh N, Yoshizawa N, Suemasu T (2015). Improved surface quality of the metal-induced crystallized Ge seed layer and its influence on subsequent epitaxy. Cryst. Growth Des..

[38] Nishida T (2019). High photoresponsivity in a GaAs film synthesized on glass using a pseudo-single-crystal Ge seed layer. Appl. Phys. Lett..

[39] Nishida T, Suemasu T, Toko K (2020). Improving photoresponsivity in GaAs film grown on Al-induced-crystallized Ge on an insulator. AIP Adv..

[40] Joy DC, Newbury DE, Davidson DL (1982). Electron channeling patterns in the scanning electron microscope. J. Appl. Phys..

[41] Imajo T, Suemasu T, Toko K (2020). Influence of grain boundaries on the properties of polycrystalline germanium. J. Appl. Phys..

[42] Kawai T, Yonezu H, Yoshida H, Pak K (1992). Ge segregation and its suppression in GaAs epilayers grown on Ge(111) substrate. Appl. Phys. Lett..

[43] Pelati D (2019). GaAs (111) epilayers grown by MBE on Ge (111): Twin reduction and polarity. J. Cryst. Growth.

